# Loss of AA13 LPMOs impairs degradation of resistant starch and reduces the growth of *Aspergillus nidulans*

**DOI:** 10.1186/s13068-020-01775-z

**Published:** 2020-08-05

**Authors:** Majid Haddad Momeni, Maria Louise Leth, Claus Sternberg, Erwin Schoof, Maike Wennekers Nielsen, Jesper Holck, Christopher T. Workman, Jakob Blæsbjerg Hoof, Maher Abou Hachem

**Affiliations:** Department of Biotechnology and Biomedicine, Technical University of Denmark, Søltofts Plads, 2800 Kgs. Lyngby, Denmark

**Keywords:** Ascomycota, *Aspergillus nidulans*, Biomass, Biorefinery, Carbohydrate oxidation, Functional genomics, Recalcitrant polysaccharides

## Abstract

**Background:**

Lytic polysaccharide monooxygenases (LPMOs) are often studied in simple models involving activity measurements of a single LPMO or a blend thereof with hydrolytic enzymes towards an insoluble substrate. However, the contribution of LPMOs to polysaccharide breakdown in complex cocktails of hydrolytic and oxidative enzymes, similar to fungal secretomes, remains elusive. Typically, two starch-specific AA13 LPMOs are encoded by mainly Ascomycota genomes. Here, we investigate the impact of LPMO loss on the growth and degradation of starches of varying resistance to amylolytic hydrolases by *Aspergillus nidulans*.

**Results:**

Deletion of the genes encoding *An*AA13A that possesses a CBM20 starch-binding module, *An*AA13B (lacking a CBM20) or both AA13 genes resulted in reduced growth on solid media with resistant, but not soluble processed potato starch. Larger size and amount of residual starch granules were observed for the AA13-deficient strains as compared to the reference and the impairment of starch degradation was more severe for the strain lacking *An*AA13A based on a microscopic analysis. After 5 days of growth on raw potato starch in liquid media, the mount of residual starch was about fivefold higher for the AA13 gene deletion strains compared to the reference, which underscores the importance of LPMOs for degradation of especially resistant starches. Proteomic analyses revealed substantial changes in the secretomes of the double AA13 gene deletion, followed by the *An*AA13A-deficient strain, whereas only a single protein was significantly different in the proteome of the *An*AA13B-deficient strain as compared to the reference.

**Conclusions:**

This study shows that the loss of AA13, especially the starch-binding *An*AA13A, impairs degradation of resistant potato starch, but has limited impact on less-resistant wheat starch and no impact on processed solubilized starch. The effects of LPMO loss are more pronounced at the later stages of fungal growth, likely due to the accumulation of the less-accessible regions of the substrate. The striking impairment in granular starch degradation due to the loss of a single LPMO from the secretome offers insight into the crucial role played by AA13 in the breakdown of resistant starch and presents a methodological framework to analyse the contribution of distinct LPMOs towards semi-crystalline polysaccharides under in vivo conditions.

## Background

The polysaccharide polymers cellulose (produced at 10^11^ t per annum) [1], chitin (similar production to cellulose) and starch (produced at 10^9^ t per annum) [2] are the most abundant renewable biomass resources on the globe. Fungal secretomes contain numerous extracellular enzymes targeting complex polysaccharides, *e.g*. lignocellulose and starch [3], both organized as insoluble semi-crystalline composites. Hydrolytic breakdown of the crystalline regions in these polymers requires the sequestering of single glycan chains from the semi-crystalline polymer matrix, which poses a large energetic penalty and lowers the efficiency of hydrolytic enzymes. Lytic polysaccharide monooxygenases (LPMOs) are copper-dependent oxidoreductases discovered in 2010 [4]. Uniquely, LPMOs catalyse the oxidative cleavage of glycosidic linkages in the crystalline regions of chitin, cellulose and starch using an exogenous reductant and H_2_O_2_ as the preferred co-substrate [5, 6]. Notably, LPMOs display synergy with hydrolytic enzymes in the degradation of chitin [4], cellulose [7] and starch [8, 9]. Currently, LPMOs are recognized as key players in recalcitrant biomass degradation and are considered as integral components in commercial enzyme cocktails targeting crystalline polysaccharides. Despite the significant industrial interest in LPMOs and recent advances in understanding their structures, mechanism and substrate-interactions [10, 11], insight is still lacking into the impact of these powerful oxidoreductases during fungal growth on semi-crystalline polysaccharides. Most functional studies on LPMOs employ a single LPMO, occasionally in the presence or absence of a hydrolase targeting the same substrate. Additionally, secretomes from distinct fungi have been shown to boost the efficiency of degradation of lignocellulose [11]. However, the contribution of individual LPMOs is not readily measurable due to the multiplicity and diversity of secreted LPMOs, other oxidoreductases and glycoside hydrolases (GHs) during growth on complex biomass polysaccharides [12, 13].

Starch is a composite of the mainly linear amylose, consisting of α-(1,4)-linked glucosyl units and the major component amylopectin, constituting up to 70–80% per weight in most plants. Amylopectin consists of α-(1,4)-linked linear glucosyl chains and 5–6% α-1,6-glucosidic branch points [14, 15]. In planta, starch is synthesized as granules that vary in size, morphology and crystallinity (15–45%). In both amylose and amylopectin, the formation of double helices by linear α-glucan chains promotes their packing into crystalline lamellae that, in amylopectin, alternate radially with amorphous regions. The A and B allomorphs are the most commonly observed in cereal and tuber starches, respectively [12]. The resistance of starch to degradation by hydrolases is strongly correlated to the details of the α-glucan packing in the granule and the crystal allomorphs. Thus, cereal starches are considered susceptible, whereas unprocessed tuber starches are considered resistant [13, 14].

**Fig. 1:**
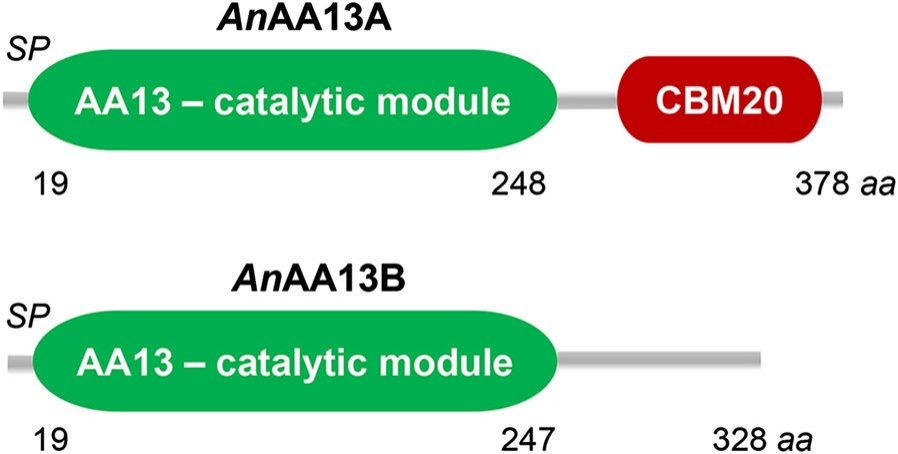
The architecture of the two starch-specific LPMOs from *A. nidulans*. Modular *An*AA13A (UniProt ID: Q5B1W7, Gene locus tag: AN5463, gene designated in this study as *aasA*) comprising an N-terminal catalytic module of AA13 and a C-terminal starch-binding module of carbohydrate binding module family 20 (CBM20) according to the CAZy database (http://www.cazy.org) and the *An*AA13B catalytic module (UniProt ID: Q5B027; Gene locus tag: AN6103, gene designated in this study as *aasB*). Both enzymes possess a predicted signal peptide (SP) consistent with their secretion

Currently, starch-active LPMOs are classified into auxiliary activity family 13 (AA13) in the CAZy database [16]. To date, AA13 LPMOs are encoded by the genomes of mainly saprotrophs and plant pathogens from Ascomycota. Frequently, but not invariantly, two AA13s are encoded in a single genome: a catalytic module (CM) and a modular enzyme comprising an N-terminal CM appended to a C-terminal starch-binding module of family 20 (CBM20) via a linker region (Fig. 1) [17]. We have previously shown that *An*AA13B was ranked among the top 10 upregulated proteins in the *Aspergillus nidulans* secretomes during growth on three different starches, while the modular *An*AA13A that is appended to a CBM20 was the most abundant in the starch-binding secretome fraction [13]. This striking abundance in the starch-induced secretomes, suggested that AA13s may play a pivotal role in starch deconstruction. The chemical simplicity of starch and presence of only two AA13s offer an appropriate model to investigate the impact of LPMOs on the deconstruction of semi-crystalline polysaccharides in vivo.

In this study, we have generated two single and a double knockout (KO) of the AA13s genes in *A. nidulans* and analysed the impact of these gene deletions on the growth and starch utilization by *A. nidulans*. The loss of AA13s, especially the enzyme with the CBM20, had a major impact on the ability of the fungus to degrade resistant potato starch. By contrast, AA13s appeared less crucial for the degradation of the less-resistant wheat starch and soluble processed potato starch. Altogether, these findings underscore the instrumental role of LPMOs, especially those appended to CBMs, in fungal breakdown of granular resistant starch and establish a framework to dissect the contribution of LPMOs in breakdown of recalcitrant polysaccharides.

## Results

### Construction of gene-deletion mutants of the starch-specific AA13 from *A. nidulans*

*Aspergillus nidulans* encodes two LPMOs of AA13 sharing 83.8% primary structure identity in their catalytic modules: *An*AA13A (AN5463, the gene is henceforth designated *aasA*, auxiliary activity family 13 specific for starch), which is the modular enzyme harbouring a C-terminal starch-binding CBM20 and *An*AA13B (AN6103, gene designated as *aasB*) (Fig. 1). To explore the significance of these LPMOs for growth on starch, a double and two individual single gene-deletion strains of the encoding *aasA* and *aasB* genes were generated. The up- and downstream sequences flanking each of the two LPMO genes were incorporated into the USER-compatible vector backbone pU2002-A containing *AfpyrG* encoding orotidine-5′-phosphate decarboxylase from *Aspergillus fumigatus* as selection marker, under the control of its native promoter and terminator flanked by a direct repeat (DR) [18]. This procedure generated the gene-deletion vectors pU2000-*aasA* and pU2000-*aasB*, which were digested to yield linear gene-targeting substrates (GTSs). The non-homologous end-joining-deficient *A. nidulans* NID1 strain [18, 19], used as a reference strain, was transformed individually with each GTS (Additional file 1: Table S1). The *aasA*Δ and *aasB*Δ deletion mutants were identified as homokaryotic deletions by diagnostic tissue-PCR, respectively, following the mutant validation scheme in Additional file 1: Fig. S1 and Table S2. To assess the effect of losing both AA13 LPMOs and their functional redundancy, we re-streaked spores of *aasB*Δ::*AfpyrG* on 5-FOA supplemented medium to select for the loss of the *pyrG marker* hereby generating a uracil and uridine auxotrophic *aasB*Δ strain (Additional file 1: Fig. S1). Protoplasts were generated from this *aasB*Δ strain to create the *aasA*Δ *aasB*Δ double-deletion strain by employing the *aasA* GTS. The resulting transformants were re-streaked and analysed by diagnostic tissue-PCR to identify the mutant strains (Additional file 1: Fig. S1). Restoration of the *argB2* allele to functional *argB* was carried out using CRISPR/Cas9 and oligo-nucleotide-mediated gene-editing [15]. A single prototrophic strain for arginine representing each of the single and double deletions was chosen for further analyses.

### Deletion of AA13 LPMO genes impairs growth and degradation of especially resistant starch by *A. nidulans*

**Fig. 2:**
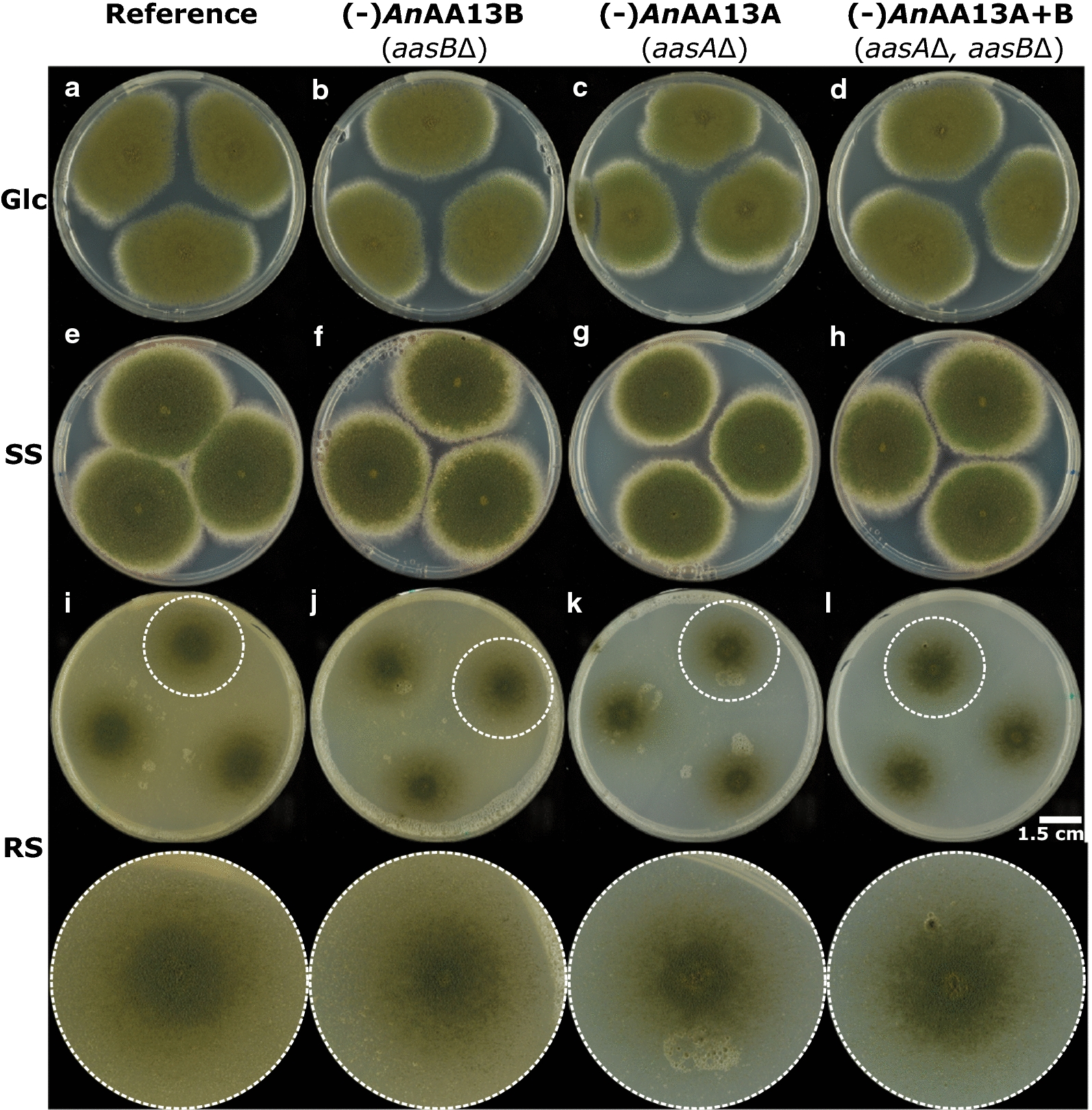
Growth phenotype of *A. nidulans* AA13 LPMO-gene deletion strains on glucose (Glc), soluble potato starch (SS) and resistant granular raw potato starch (RS). Each plate is inoculated from a liquid conidia solution (1 × 10^6^ spores mL^−1^) at three positions. **a**–**d***A. nidulans* strains grown on Glc for 7 days, **e**–**h***A. nidulans* strains grown on SS for 7 days, and **i**–**l***A. nidulans* strains grown on RS for 8 days. White circles indicate the enlarged colonies in the bottom panel

To evaluate the impact of the AA13s, we used three different starches: (1) raw granular potato starch, (2) wheat granular starch and (3) processed soluble potato starch. The raw potato starch comprises large (up to 100 µm in diameter) granules of the tightly packed B-type allomorph, considered as a resistant starch type, as compared to the A-type allomorph of the smaller cereal (including wheat) granules that are considered more susceptible to amylolytic hydrolases [13, 14, 16]. The processed potato starch is a soluble hydrated form (granular structure is disrupted), which is highly accessible to α-amylases. The *A. nidulans* reference and AA13 gene deletion strains were grown for up to 9 days on minimal media agar plates supplemented with glucose, soluble potato starch, or resistant potato starch, which has been thoroughly washed as previously described [17] to minimize the presence of soluble easily accessible residues to fungal α-glucan hydrolases. The growth phenotype of the reference and AA13 gene deletion strains was not distinguishable on glucose (Fig. 2a–d, Additional file 1: Fig. S2). The phenotype of the mutants was also rather similar to the reference strain on soluble potato starch (Fig. 2e–h, Additional file 1: Fig. S3). Strikingly, the strains lacking AA13s showed a clearly distinguishable growth phenotype on resistant potato starch, as compared to the reference (Fig. 2, Supplementary file Fig. S4). Remarkably, this difference was not readily visible during the early growth phase (days 1–5), but became evident from day 6, as the radial growth rings were smaller and less regular. The (-)*An*AA13B strain displayed the least severe phenotype, whereas the growth was most drastically affected for (-)*An*AA13A and the double KO (-)*An*AA13A + B (Fig. 2i–l, Additional file 1: Fig. S4).

These results revealed a significant difference in the utilization of resistant starch by the (-)*An*AA13 strains, especially those lacking the starch-binding *An*AA13A on.

**Fig. 3:**
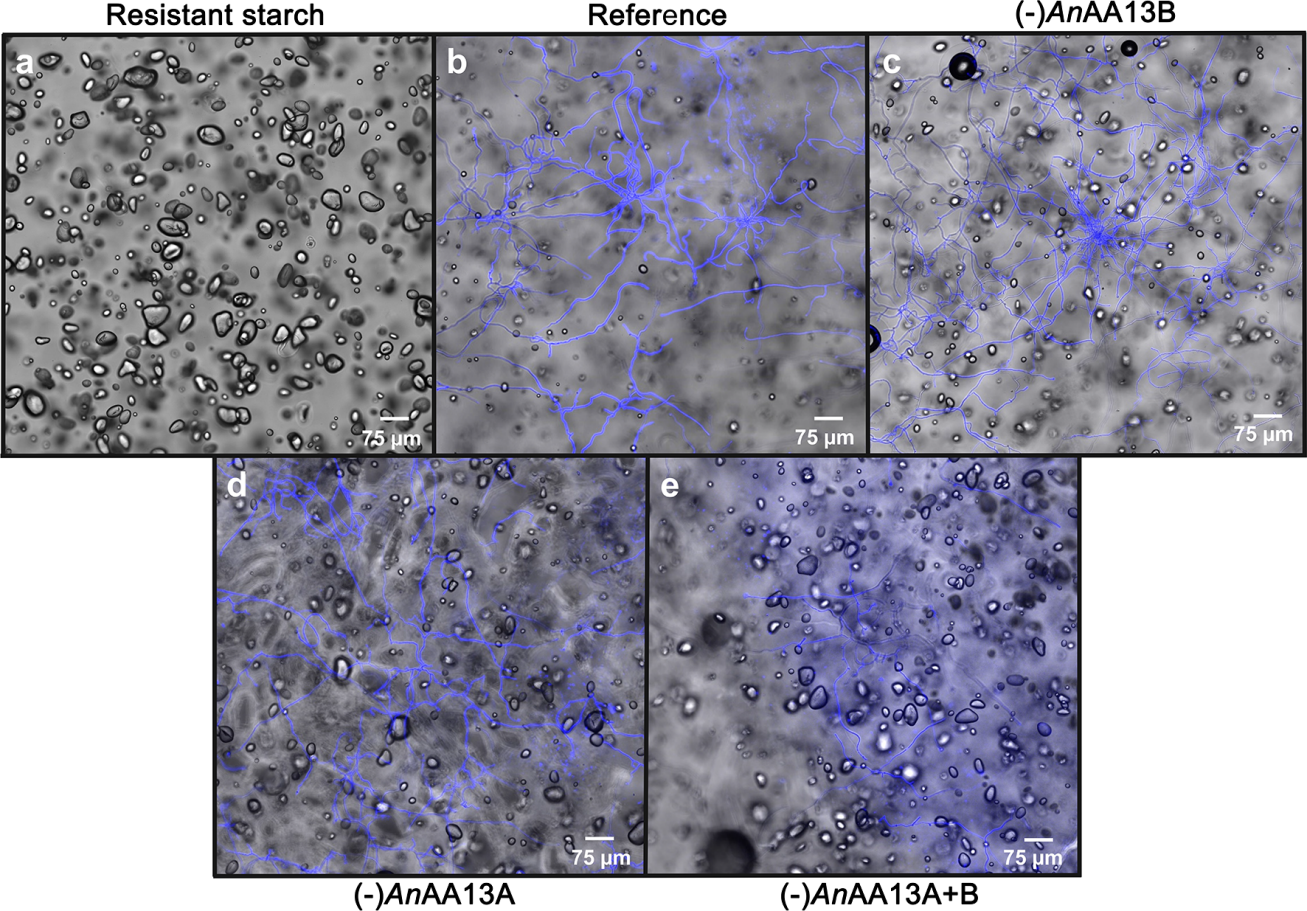
Confocal laser scanning microscopy images of *A. nidulans* reference and AA13 gene deletion strains grown on resistant potato starch. The analysis was performed on an agar minimal medium supplemented with granular starch for 20 h. **a** Starch with no fungal inoculum (control), **b** reference *A. nidulans* strain, **c***An*AA13B deletion strain, **d***An*AA13A deletion strain and **e** double AA13 gene deletion strain. The fungal hyphae are visualized by calcofluor white (CFW) staining (blue). The micrographs of the deletion strains are arranged based on the severity of the starch degradation phenotype, based on size and number of residual starch granules in the focal plane

To provide a rationale for the observed phenotypes, we used confocal laser scanning microscopy (CSLM) to analyse growth on the same resistant potato starch. The analysis was performed in a thin agar medium layer supplemented with resistant starch on a microscope slide for 20 h. The relatively short incubation period was set to avoid artefacts related to the drying the thin solidified medium layer and extensive hyphal growth. This analysis revealed that the number and size of granules in the reference strain culture were greatly reduced consistent with efficient degradation of the resistant starch (Fig. 3a, b).

**Fig. 4:**
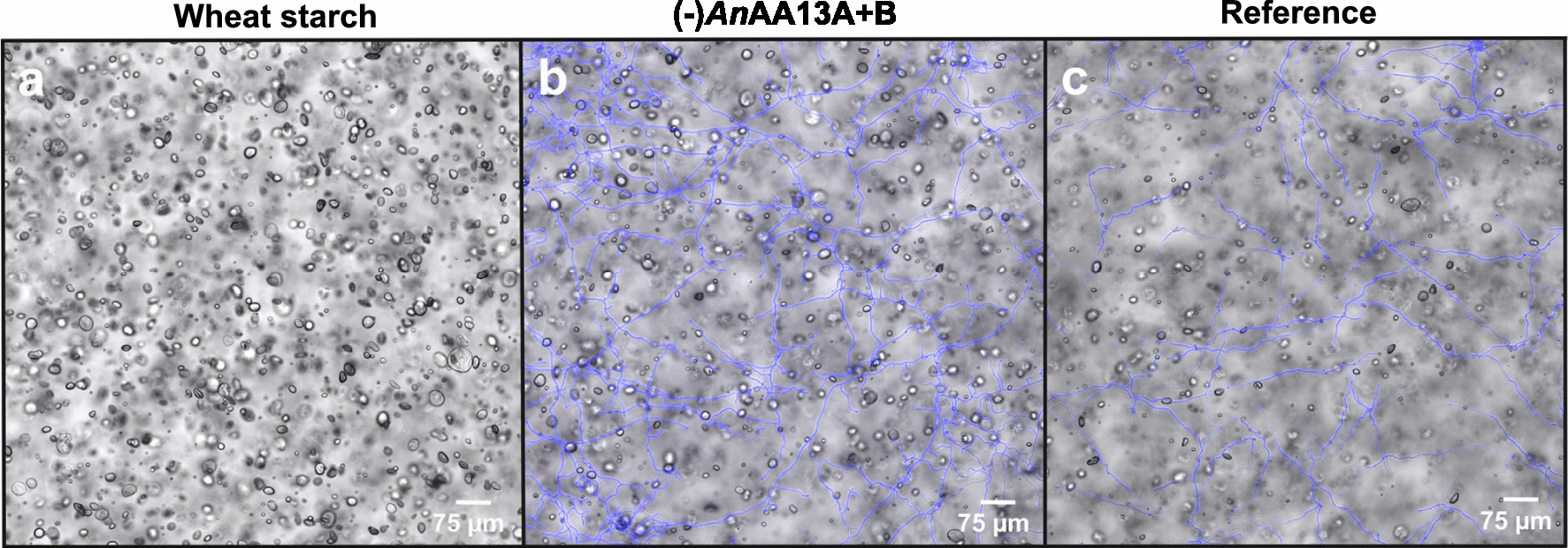
Confocal laser scanning microscopy images of *A. nidulans* reference and the double AA13 gene deletion strain grown on an agar minimal medium supplemented with wheat starch for 20 h. **a** Wheat granular starch, as a control. **b** (-)*An*AA13A + B and **c** reference *A. nidulans* strains. The fungal hyphae are visualized by calcofluor white staining (blue)

The lack of *An*AA13B lowered the efficiency of resistant starch utilization as evident from the larger number and average size distribution of residual starch granules as compared to the reference strain (Fig. 3a–c). However, the least reduction in the size and amount of starch granules, compared to the starting material, was observed for the (-)*An*AA13A and the double (-)*An*AA13A + B strains (Fig. 3a, d, e). Thus, the ability of *A. nidulans* to degrade resistant granular starch appeared to be dependent on the presence of the AA13s, especially *An*AA13A that harbours the CBM20. To determine the effect of AA13 deficiency on a more susceptible granular starch, a similar analysis including the reference and the (-)*An*AA13A + B strain, was performed on wheat starch, which has been shown to sustain excellent growth and abundant secretion of AA13s by *A. nidulans* in our previous analysis [18]. Interestingly, the impact of the AA13s deficiency was clearly observable based on the larger number of residual wheat starch granules observed for the (-)*An*AA13A + B strain as compared to the reference (Fig. 4). Nonetheless, the impairment in starch degradation was less severe for wheat starch as compared to resistant potato starch based on the marked reduction in the amount and size of granules in the (-)*An*AA13A + B strain, compared to the starting material (Figs 3a, e and 4a, b).

### Resistant starch degradation is impaired in liquid cultures of AA13 LPMO gene KOs

**Fig. 5:**
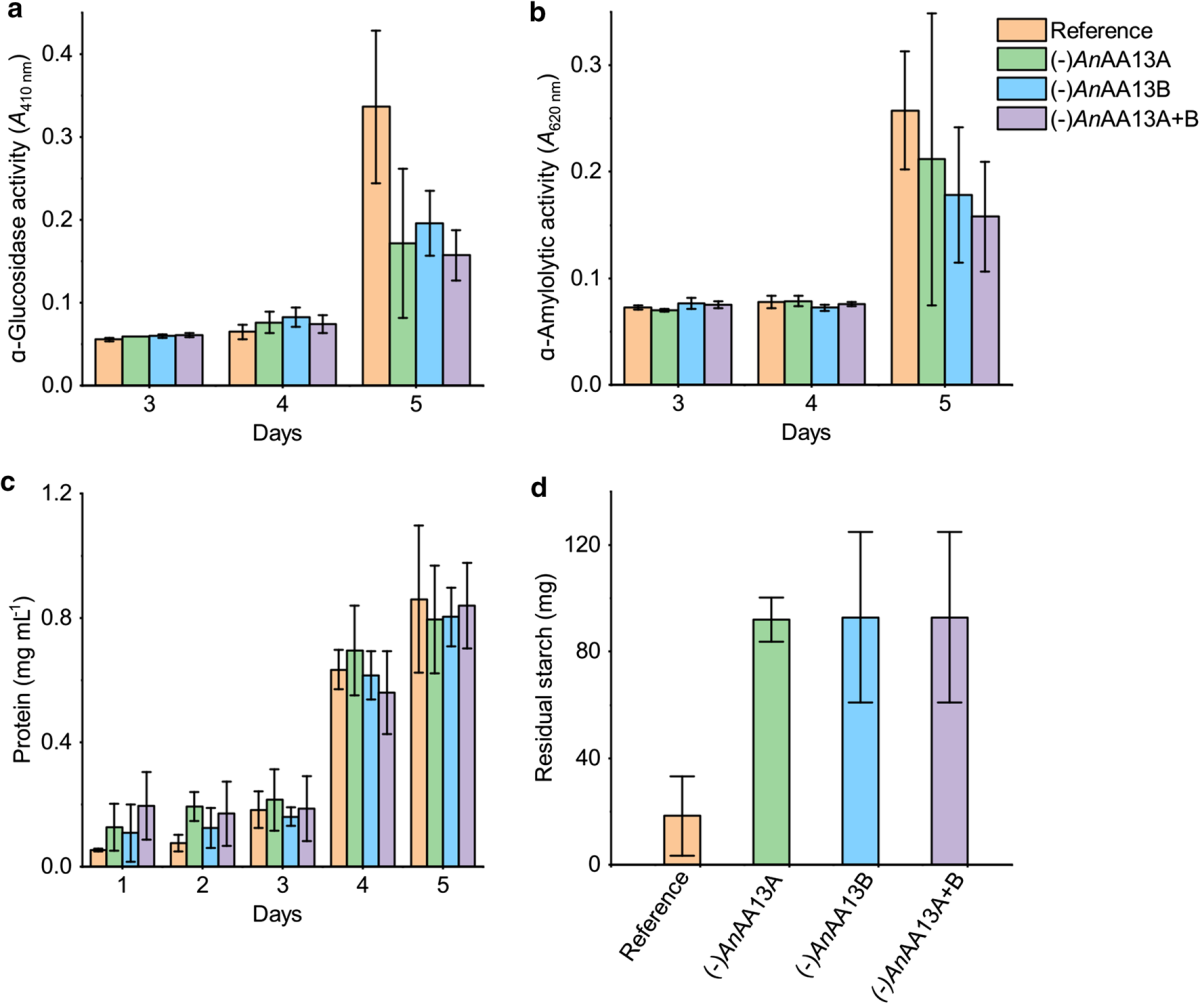
Analysis of hydrolytic starch-degrading activities, secreted protein levels and residual starch in reference and AA13 gene deletion strains grown in liquid medium on resistant potato starch. **a** α-Glucosidase and **b** α-amylolytic activities in culture supernatants as analysed using *para* nitrophenyl α-glucopyranoside and insoluble cross-linked blue starch, respectively. **c** Protein concentrations in the culture supernatant. **d** The residual insoluble starch in the harvested culture suspension (day 5). The start starch concentration in each culture was 0.5 g

We performed a growth experiment on the same resistant potato starch in liquid media to allow for comparative biochemical and proteomic analyses of the AA13 loss. The culture supernatants of the reference and the gene-deletion strains were analysed for the levels of α-amylolytic and α-glucosidase activities that mediate the degradation of starch and depolymerization of solubilized maltodextrins to glucose, respectively. The levels of these activities were similar, with a trend of modest decrease in the activities of the gene-deletion strains at day 5 (Fig. 5a, b). The protein concentrations in the culture supernatants were also analysed with only modest differences between the reference and AA13 deficient strains (Fig. 5a, b). By contrast, approximately fivefold higher residual starch content was estimated in the AA13 deletion strain cultures compared to the reference strain.

We also compared the biomass in the harvested culture using the mannose content and the wet weight of the retentate form the filtered culture suspension. The cell wall of *Aspergillus* contains galactomannan [20] as a structural component, which is not present in starch as opposed to glucose, thus it was used as a proxy for fungal biomass formation. Based on these analyses, the changes were modest between the strains, with the direct wet-weight measurement giving a slight reduction of biomass for the gene-deletion strains compared to the reference (Additional file 1: Fig. S5).

### Proteomics analysis of the AA13 KOs and reference strains

To bring insight into the impact of the AA13 gene deletion on *A. nidulans* growth on resistant potato starch, the secretomes of the reference, both the single and the double AA13 gene deletion strains were compared at day 3 and 5 of growth in the liquid culture (Additional file 2). The analysis identified 816 proteins, but strikingly no significant changes of any of these proteins were observed between the reference and AA13 deletion strains at day 3. Principal component analysis of the data showed that the datasets from the reference and the AA13 deletion strains are clustered together at day 3, but not on day 5, especially for the (-)*An*AA13A + B datasets that formed a distinct cluster (Additional file 1: Fig. S6). For (-)*An*AA13B, only a hypothetical protein (UniProt: Q5AVZ4) also observed in the (-)*An*AA13A + B, was statistically regulated relative to the reference (Additional file 2). This protein showed distant homology to protein annotated as *Schizosaccharomyces pombe* stress response component (UniProt: P87179.3).

**Fig. 6:**
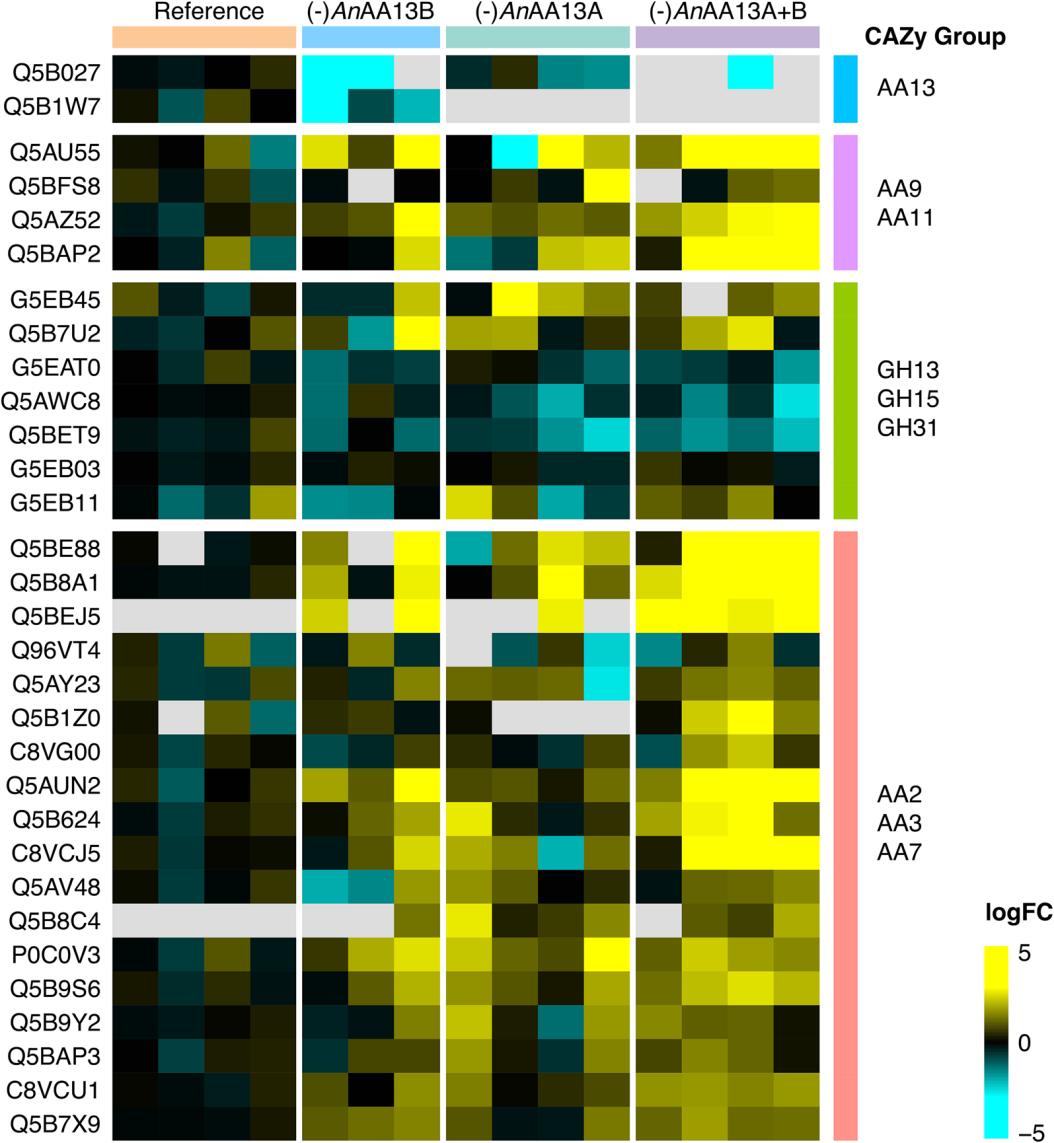
Heat map comparison of the CAZy families identified in the secretomes of reference and AA13 gene deletion *A. nidulans* strains at day five of growth on resistant potato starch

By contrast, the secretomes of (-)*An*AA13A and (-)*An*AA13A + B revealed 14 and 204 (37 CAZymes) significantly regulated proteins (Log_2_FC > 2, P ≤ 0.05), respectively (Fig. 6, Additional file 1: Fig. S7, Additional file 2). Only one of the seven amylolytic hydrolases (GH31, AgdE, UniProt Q5BET9) was amongst the 20 regulated GHs for the (-)*An*AA13A + B dataset (Fig. 6, Additional file 2).

Interestingly, this enzyme was downregulated in this dataset, suggestive of a limited access to the oligomers that mediate the transcriptional upregulation. Hypothetical proteins, proteases and a considerable proportion of intracellular proteins were amongst the upregulated proteins, which suggests a distinctly different physiological state of the (-)*An*AA13A + B compared to the reference.

## Discussion

The action of LPMOs, their synergy with other hydrolases [21, 22] and fungal oxidoreductases [4, 23] have been extensively investigated in vitro. LPMOs are proposed to render their substrates more accessible to the action of hydrolases, as also supported by recent studies showing the role of LPMOs in the disruption of the structure of cellulose fibres [24]. Insight into the actual contribution of distinct LPMOs against more complex backgrounds resembling such secretomes is lacking to date. This study uniquely investigates the impact of AA13 genes on the growth of *A. nidulans* on starches with varying degree of resistance to enzymatic hydrolysis.

The visual phenotypic difference observed for the AA13 gene deletion strains indicated a marked change in growth on resistant potato starch on solid media (Fig. 2). By contrast, these strains grew similarly to the reference on glucose and soluble potato starch, suggesting that the observed AA13-associated phenotypic changes were specific to resistant potato starch with an intact granular structure (Figs. 2, 3, 4, Additional file 1: Fig. S2–S5). An extent of growth on the resistant starch substrate is expected, as all gene deletion strains retain an amylolytic enzyme repertoire that comprises 12 enzymes. Indeed seven of the eight amylolytic hydrolases that have been identified in the secretomes of *A. nidulans* in our previously reported study [13] were detected in the present study (Additional file 2). α-Amylolytic and α-glucosidase activities were also detected in the AA13 gene deletion strains (Fig. 3). The modest changes in measured activities are supported by the proteomic analyses revealing a slight down-regulation trend of most amylolytic hydrolases (GH13 α-amylases, GH31 α-glucosidases and GH15 glucoamylases) in the AA13-deficient strains on day 5 (Fig. 6, Additional file 2). The amylolytic hydrolases are likely to preferentially mediate breakdown of less-resistant sites, consistent with the similar growth between the reference and AA13-deficient strains on processed soluble potato starch, where the granular organization has been disrupted (larger extent of α-glucan chains are hydrated). By contrast, the depolymerization of the granular form of potato starch is greatly impaired, when one or both of the AA13s is lost.

Our findings highlight a paramount role of the modular starch binding *An*AA13A in the degradation of resistant starch, which is supported by: (1) the growth phenotype on solid media (Fig. 2); (2) the microscopic analysis showing a trend of increased amount and size of residual starch granules in (-)*An*AA13B, followed by (-)*An*AA13A and (-)*An*AA13A + B as compared to the reference strain (Fig. 3); (3) the secretomic analyses showing only a single significantly regulated protein in the secretomes of (-)*An*AA13B and the reference strain as opposed to large changes in the secretomes of the (-)*An*AA13A and (-)*An*AA13A + B strains (Fig. 6, Additional file 2). These observations support the notion that *An*AA13A is partly complementing the loss of *An*AA13B in the (-)*An*AA13B, which does not apply to the same extent for the (-)*An*AA13A strain. Only about a fifth of the significantly regulated proteins in the secretomes of the (-)*An*AA13A + B strain at day 5 compared to the reference were CAZymes. Thus, the majority were proteins with other functions including proteases and intracellular stress proteins (Additional file 2). This is indicative of large changes in the physiological state of the fungus in the double (-)*An*AA13A + B strain and/or increased extent of lysis of fungal hyphae due to stress (starvation).

A deleterious effect on the activity of a bacterial modular cellulose-specific LPMO as a result of deleting the appended CBM has been reported [25]. In another fungal cellulose-specific LPMO, the deletion of the CBM also reduced the yield of saccharification, but was not associated with a similar impairment of substrate degradation as above, suggesting that the extent of CBM contribution maybe specific for the enzyme–substrate combination [26]. The abundant secretion of both AA13 LPMOs from *A. nidulans* on wheat, maize and pea starches observed in a previous study [13], demonstrated that AA13s are universally deployed for the degradation of different starches and secondly that these enzymes may exhibit functionally different properties. *An*AA13A displayed moderate affinity to the starch mimic β-cyclodextrin (*K*_d_ ≈ 40 µM), which is mediated by the CBM20 starch-binding module [27]. This affinity for β-cylcodextrin (and starch) is not provided by the active site, as the *An*AA13B homologue lacking the CBM20 was not captured by the β-cyclodextrin affinity column [13]. The active site of AA13 LPMOs has been proposed to recognize the helical conformation of starch in a shallow groove [7, 28], albeit likely with a much lower affinity than the CBM20-mediated binding as argued above. A possible advantage of the *An*AA13B is that the activity is less localized due to the more dynamic lower affinity binding and smaller size as compared to *An*AA13A, which may allow deeper penetration in the starch granule. This feature, maybe more important in cereal starches, especially maize which has a channel structure [19]. A model of inside out degradation mechanism has been proposed for such cereal starches, whereas an exo-corrosion model has been suggested for B-type tuber resistant starches [20], where the starch binding functionality of *An*AA13A is likely to be more important than the smaller size of *An*AA13B. Taken altogether, this study demonstrates a crucial role of LPMOs in the degradation of resistant starch, and possibly other semi-crystalline substrates.

## Conclusions

The study provides evidence that the deletion of one or both AA13s reduces the degradation efficiency of granular resistant starch, but not processed solubilized starch. This impact of the AA13 deficiency is less pronounced on wheat starch that is more susceptible to degradation by hydrolytic enzymes. This study offers a framework to assess the contribution of distinct LPMOs in fungal secretomes on the utilization of specific semi-crystalline polysaccharides.

## Methods

### Carbohydrates and chemicals

Commercial granular raw resistant potato starch was from Bauckhof (Rosche, Germany), soluble potato starch (S2630) and granular unmodified wheat starch (S5127) were from Sigma-Aldrich (St. Louis, USA). The granular starches for the liquid or solid media growth experiments were not autoclaved to minimize changes of the native starch granule structure, but the soluble starch comprising was autoclaved and used for the growth phenotype assay on plates. The granular starches were thoroughly washed four times with five consecutive volumes of Milli-Q water, 70% ethanol, Milli-Q water and 70% ethanol at 30 °C with intermittent centrifugation at 5000x*g*. The residual ethanol was evaporated in a laminar flow bench and each starch substrate was subsequently resuspended in autoclaved growth media and added to make a homogenous suspension in agar plate before solidification. *p*-Nitrophenyl-α-d-glucopyranoside (PNPG) was from Sigma-Aldrich, and insoluble Blue Starch (iBS) was a custom preparation from Pharmacia (Uppsala, Sweden).

### Strains and media

The *An*AA13A (Gene locus tag: AN5463 (gene designated *aasA* in this study), UniProt ID: Q5B1W7) and *An*AA13B (Gene locus tag: AN5463 (*aasB* in this study), UniProt ID: Q5B1W7) starch-specific LPMO encoding genes were deleted in the present work. *A. nidulans* strain NID1 (*argB2, pyrG89, veA, nkuA*Δ) [29] was used as a reference strain for gene deletions. This strain was amenable to efficient genetic manipulation by deficiency for NHEJ (non-homologous end joining) and two auxotrophies, but otherwise resembled wild-type *A. nidulans* strains with regard to ability to utilize complex glycans as carbon source. The *argB* and *pyrG* encode ornithine carbamoyltransferase and orotidine-5′-phosphate decarboxylase activities, respectively. The *argB2* mutant was corrected to functional *argB* in the final mutant strains using CRISPR/Cas9 and the oligonucleotide mediated gene-editing strategy as described recently [30]. The *pyrG89* mutation was complemented by the use of the heterologous *AfpyrG* from *Aspergillus fumigatus.* This gene, which was extended by its native promoter and terminator and flanked by a direct repeat (DR) of 290 bp [29], was incorporated into the gene-targeting substrates. Genomic DNA (gDNA) from NID1 was isolated using FastDNA SPIN Kit for Soil DNA extraction kit (MP Biomedicals, USA). All strains employed in this study are listed in Additional file 1: Table S1. *Escherichia coli* strain DH5α was used as host for plasmid propagations.

*Aspergillus nidulans* was cultivated on solid and liquid glucose based minimal medium (MM) containing (per litre): 1% glucose (w/v), 6 g NaNO_3_, 0.52 g KCl, 0.52 g MgSO_4_·7H_2_O, 1.52 g KH_2_PO_4_ [18], 0.001% (w/v) thiamine, and 1 mL of trace elements [31], 2% (w/v) agar for solid medium. For the generation of the gene deletion strains, this medium was supplemented with 10 mM uridine (Uri), 10 mM uracil (Ura), and/or 4 mM l-arginine (Arg). Solid plates containing 5-fluoroorotic acid (5-FOA, 1.3 mg mL^−1^) were made from MM + Arg + Uri + Ura for counter-selecting *pyrG*. The glucose was replaced by 1 M sucrose for transformation media (TM). For protoplasting, 100 mL of liquid MM + arg + Ura + Uri in unbaffled shake flasks were inoculated to a concentration of 1 × 10^6^ conidia mL^−1^, and incubated at 37 °C with stirring at 150 rpm for 20 h.

### Gene-deletion vector and fungal strain generation

All PCR products used for cloning were amplified by the proof-reading PfuX7 polymerase [32] on an Agilent sure cycler (Agilent, Santa Clara, CA) using standard PCR programs: 98 °C, 2 min; 35 cycles (98 °C, 15 s; 62 °C, 30 s; 72 °C, 3 min); 72 °C, 5 min. The standard reaction volumes were 50 µL including 1 × Phusion^®^ HF Buffer (New England Biolabs (NEB), USA), 0.2 mm dNTPs, 0.4 µM primers (Integrated DNA Technologies, Belgium), 1 U PfuX7, < 10 ng of gDNA, 3% DMSO. PCR products were purified from agarose gels using a GFX purification kit (GE Healthcare, Chicago, USA). All vectors were assembled by USER cloning as described previously [19]. The plasmid´s linearization reaction were performed using SwaI (NEB) according to manufacturer’s instructions and terminated by heat inactivation (65 °C for 20 min). Protoplastation and transformation were performed in *A. nidulans*, respectively, as described before [33, 34]. Transformants were restreaked at least twice, and analysed rigorously by diagnostic tissue-PCR as described elsewhere [34], following the validation scheme, depicted in Additional file 1: Fig. S1.

### Growth phenotype assay on solid medium

The reference *A. nidulans* and the gene deletion strains derived thereof were cultivated on solid MM at 30 °C for 5 days. Conidia were gently harvested using ice-cold sterilized 0.9% (w/v) NaCl solution to a final concentration of 1 × 10^8^ conidia mL^−1^, which was used to inoculate plates with the same medium containing 1% (w/v) or Glc, soluble potato starch or resistant potato starch at three positions. To minimize bacterial contamination of the plates with the washed non-autoclaved resistant potato starch, ampicillin (100 µg mL^−1^) and chloramphenicol (34 µg mL^−1^) were added to the medium together with the starch before solidification. Growth was monitored for up to 9 days.

### Confocal laser scanning microscopy (CLSM)

For the CLSM analysis, 700 µL MM containing 1% agar and either potato or wheat starch (1% w/v) as a sole carbon source, and supplemented with antibiotics as described above, were added as 1.5 × 4 cm square to sterilized microscope glass slides. After 30 min solidification, the media were inoculated by adding 10 µL of 1 × 10^5^ mL^−1^ conidia suspension. Prior to mounting the cover glass onto the incubated slides (with and without inoculum), two drops of 3 µL tenfold diluted 0.1% (w/v) calcofluor white (CFW, Sigma-Aldrich) in Milli-Q were added to the agar with 1 cm distance between the drops. The microscopy slides were placed in petri dishes stacked in micro-perforated plastic bags at 37 °C for 20 h. The slides were assembled by a 50 × 24 mm cover glass #1 (Knittel AG, Braunschweig, Germany) and incubated at room temperature for an hour prior microscopy analysis. The assembled slide-samples were mounted up-side-down on a Leica Microsystems SP8 confocal microscope (Leica, Mannheim, Germany). Images were alternatively captured using a 10× HC Plan Fluotar objective, or a 63× HC N Plan-Apochromat oil immersion objective. Cells stained with CFW were imaged in confocal mode, using 405 nm excitation and 480–545 nm emission detection. Transmitted light images were acquired via a non-confocal configuration mode using a 552 nm laser as illumination. CFW has optimal excitation and emission wavelengths below what were used here, but due to technical limitations, the indicated wavelengths still captured clear images of the fungal cell components.

### Growth experiments in liquid medium

*Aspergillus nidulans* and the gene-deletion mutants were grown in 250 mL baffled Erlenmeyer flasks containing 50 mL MM containing 1% (v/w) resistant potato starch as a carbon source. The MM was supplemented with ampicillin (100 µg mL^−1^) and chloramphenicol (34 µg mL^−1^) to minimize bacterial contamination. Each flask was inoculated to a final concentration of 1 × 10^6^ spores mL^−1^ and cultivated on a rotary shaker (150 rpm) for 5 days at 30 °C. The growth was performed in four biological replicates.

### Biomass, residual starch, amylolytic activities and protein analyses of liquid cultures

Filtration through a cloth, was used to separate the final fungal biomass (retentate) from the culture suspension. The wet weight was determined and the biomass was frozen at − 20 °C until further use. The mannose content of the final fungal biomass was determined by acid hydrolysis. The biomass (from 40 mL suspension) was thawed, transferred to Teflon-capped pressure tubes, freeze dried, suspended in 1.5 mL 72% (w/v) H_2_SO_4_ and incubated at 30 °C, 1 h. The samples were immediately cooled down and stored at 5 °C until further analysis. The mannose content in the samples was quantified using an HPAEC-PAD system equipped with PA1 column and post-column NaOH addition as described previously [35].

To separate the residual insoluble resistant potato starch, the culture suspension (filtrate from the above step) was centrifuged at 5000×*g* for 30 min. The pelleted starch was washed with twice 96% ethanol, re-centrifuged as above. The ethanol was removed and the starch dried prior to determining the weight.

For enzyme activity measurements, 500 µL culture supernatant was concentrated 50-fold and buffer exchanged to assay buffer (10 mM MES, 0.005% Triton X-100, pH 6.5) using Amicon^®^Ultra Centrifugal Filters (Merck Millipore, Cork, Ireland).

α-Amylase activity of the fungal cultures was assayed toward insoluble Blue Starch (iBS, 6.25 mg mL^−1^) suspended in assay buffer. The reaction mixture (180 μL iBS + 20 μL supernatant) was incubated for 20 h at 37 °C, stopped by 0.5 M NaOH (40 μL), centrifuged (20,000×*g*, 3 min), and 200 μL supernatant was transferred to a 96 well microtiter plate and the absorbance read at 620 nm. α-Glucosidase activity was determined towards 2 mM *p*-nitrophenyl-α-d-glucopyranoside (PNPG) in assay buffer. The reaction mixture (40 μL PNPG and 10 μL supernatant) was incubated for 20 h at 37 °C, stopped by adding 1 M Na_2_CO_3_ (200 μL), centrifuged (20,000×*g*, 3 min). Then, 200 μL supernatant was transferred to a 96 well microtiter plate and the absorbance read at 410 nm.

The protein concentration was determined using Pierce™ Rapid Gold BCA Protein Assay Kit (Thermo Fisher Scientific, Waltham, USA) according to the manufacturer´s instruction using bovine serum albumin as standard. Amylolytic activities protein concentration were measured in the four biological replicates and presented as the means with standard deviations.

### Proteomics

Samples (2 mL) from four biological replicate cultures were collected from day 3 to 5 under sterile conditions. The samples were centrifuged (20,000×*g*, 10 min, 4 °C) and filtered (0.2 μm regenerated cellulose syringe filters, Thermo Fisher Scientific) to remove mycelia and residual insoluble starch. The filtrate was flash frozen in liquid nitrogen and stored at − 80 °C until further use. Proteins of the culture filtrates were precipitated by addition of acetone to a final concentration of 80% (v/v), incubation over night at − 20 °C, and centrifugation (4000×*g,* 10 min) to pellet the precipitated protein. The protein pellets were dissolved in 50 µL buffer (6 M guanidinium hydrochloride, 10 mM Tris(2-carboxyethyl)phosphine hydrochloride, 40 mM 2-chloroacetamide, 50 mM HEPES pH 8.5), boiled (5 min 95 °C) and sonicated (5x60 s at 4 °C) (Bioruptor, Diagenode). For digestion, 45 µL sample were diluted 1:3 in digestion buffer (50 mM HEPES, 10% (v/v) acetonitrile (ACN), pH 8.5) and incubated with 200 ng LysC (MS grade, Wako) at 37 °C for 3 h. Subsequently, the samples were diluted 1:10 in digestion buffer and further digested with 200 ng trypsin (MS grade, Promega) at 37 °C for 18 h. The digestion was stopped by addition of 450 µL 2% trifluoroacetic acid (TFA). Samples were desalted using SOLAµ HRP plate (Thermo Fisher Scientific) by activation with 200 µL methanol and 200 µL MS buffer (80% ACN, 0.1% formic acid), equilibration 2 × 200 µL 3% ACN, 1% TFA, sample application, 2x wash with MS buffer A (0.1% FA) and elution with 2× 300 µL 40% ACN, 0.1% FA. Between each solvent application, the plate was centrifuged (1500 rpm, 1 min). Eluted peptides were dried in an Eppendorf Speedvac (3 h at 60 °C) and reconstituted in 2% (v/v) ACN, 1% (w/v) TFA acid prior to mass spectrometry (MS) analysis. The peptides were loaded on the mass spectrometer by reverse phase chromatography through an inline 15 cm C18 column (Thermo EasySpray ES804A) connected to a 2 cm long C18 trap column (164705, Thermo Fisher Scientific) using a Thermo Easy nLC 1200 HPLC system. Peptides were eluted with a mobile phase of constant 0.1% (w/v) FA at 250 nL min^−1^ over 100 min with a five step gradient of buffer B (80% (v/w) CAN, 0.1% FA): 0–68 min gradient of 6–23%, 68–85 min 23–38%, 85–90 min 38–60%, 90–93 min 60–95%, and 93–100 min 95%. Analysis was performed on a Q-Exactive HF-X instrument (Thermo Fisher Scientific) run in a data-dependent manner using a “Top 15” method. Full MS spectra were collected at 60,000 resolution, with an AGC target set to 3 × 10^6^ ions or maximum injection time of 50 ms. Peptides were fragmented via higher-energy collision dissociation (normalized collision energy = 25). The intensity threshold was set to 1 × 10^4^, dynamic exclusion to 60 s and ions with a charge state < 2, > 6 or unknown species were excluded. MS/MS spectra were acquired at a resolution of 15,000, with an AGC target value of 1 × 10^5^ ions or a maximum injection time of 60 ms. The scan range was limited from 350 to 1850 *m/z*.

The raw files were analysed using Proteome Discoverer 2.4. Label-free quantitation (LFQ) was enabled in the processing and consensus steps, and spectra were matched against the *A. nidulans* database obtained from Uniprot (UniProt ID: UP000000560).

Dynamic modifications were set as Oxidation (M), Deamidation (N,Q) and Acetyl on protein N-termini. Cysteine carbamidomethyl was set as a static modification. All results were filtered to a 1% FDR, and protein quantitation done using the built-in Minora Feature Detector.

The normalized abundance values for each sample were then log_2_ transformed and used for further hypothesis testing. A linear modelling approach (LIMMA v3.40.6) was applied using the R statistical computing environment (v3.6.1). After a principal component analysis of the protein abundances from day 3 and day 5 samples, the largest source of variability was observed to be related to the sampling day, and that day 3 samples showed little effects due to the strain background. For this reason, only day 5 samples were used in the statistical model. A linear model was applied where log2-abundance values were used to estimate the parameters for each strain background and comparisons (contrasts) were made for each KO strain relative to the reference strain. Empirical Bayes statistics were used to estimate false discovery rates (*q*) using the Benjamini–Hochberg method for each comparison. The difference in the group means is represented by the log_2_-fold change (logFC) from the reference. The mass spectrometry proteomics data have been deposited to the ProteomeXchange Consortium via the PRIDE [21] repository under the entry PXD020101.

## Supplementary information

**Additional file 1: Table S1.** List of strains used in the study. **Table S2.** Primers for generation and validation of the *A. nidulans* mutant strains. **Fig. S1.** Gene targeting and mutant-strain validation. **Fig. S2.** Growth of reference and AA13 gene deletion strains on glucose. **Fig. S3.** Growth of reference and AA13 gene deletion strains on soluble potato starch. **Fig. S4.** Growth of reference and AA13 gene deletion strains on resistant potato starch. **Fig. S5.** Biomass analysis of reference and AA13 gene deletion strains on resistant potato starch at day 5. **Fig. S6.** Principal component analysis of the proteomic data sets during growth on resistant starch. **Fig. S7.** Venn diagram of the significantly upregulated proteins during growth on resistant potato starch.

**Additional file 2:** Label-free Proteomics.

## Data Availability

The *A. nidulans* gene-deletion strains are available at DTU Bioengineering. The MS proteomics data have been deposited to the ProteomeXchange Consortium with the dataset identifier PXD020101.

## Abbreviations

AA: Auxiliary activity enzyme
CAZy: Carbohydrate-active enzyme
CBM: Carbohydrate-binding module
CFW: Calcofluor white
CLSM: Confocal laser scanning microscopy (CLSM) analysis
KOs: Knock-outs
LPMO: Lytic polysaccharide monooxygenase
MM: Minimal media
NHEJ: Non-homologous end joining
SS: Soluble potato starch
RS: Resistant potato starch

## References

[1] Cragg SM, Beckham GT, Bruce NC, Bugg TDH, Distel DL, Dupree P (2015). Lignocellulose degradation mechanisms across the Tree of Life. Curr Opin Chem Biol.

[2] Bouws H, Wattenberg A, Zorn H (2008). Fungal secretomes - Nature’s toolbox for white biotechnology. Appl Microbiol Biotechnol.

[3] Vaaje-Kolstad G, Westereng B, Horn SJ, Liu Z, Zhai H, Sørlie M (2010). An oxidative enzyme boosting the enzymatic conversion of recalcitrant polysaccharides. Science.

[4] Eijsink VGH, Ludwicka K, Felice AKG, Preims M, Ludwig R, Breslmayr E (2016). Extracellular electron transfer systems fuel cellulose oxidative degradation. Science.

[5] Bissaro B, Røhr ÅK, Müller G, Chylenski P, Skaugen M, Forsberg Z (2017). Oxidative cleavage of polysaccharides by monocopper enzymes depends on H_2_O_2_. Nat Chem Biol.

[6] Frommhagen M, Sforza S, Westphal AH, Visser J, Hinz SWA, Koetsier MJ (2015). Discovery of the combined oxidative cleavage of plant xylan and cellulose by a new fungal polysaccharide monooxygenase. Biotechnol Biofuels.

[7] Lo Leggio L, Simmons TJ, Poulsen JCN, Frandsen KEH, Hemsworth GR, Stringer MA (2015). Structure and boosting activity of a starch-degrading lytic polysaccharide monooxygenase. Nat Commun..

[8] Forsberg Z, Vaaje-kolstad G, Westereng B, Bunsæ AC, Stenstrøm Y, Mackenzie A (2011). Cleavage of cellulose by a cbm33 protein. Protein Sci.

[9] Agger JW, Isaksen T, Várnai A, Vidal-Melgosa S, Willats WGT, Ludwig R (2014). Discovery of LPMO activity on hemicelluloses shows the importance of oxidative processes in plant cell wall degradation. Proc Natl Acad Sci USA..

[10] Beeson WT, Vu VV, Span EA, Phillips CM, Marletta MA (2015). Cellulose degradation by polysaccharide monooxygenases. Annu Rev Biochem.

[11] Coutinho PM, Andersen MR, Kolenova K, vanKuyk PA, Benoit I, Gruben BS (2009). Post-genomic insights into the plant polysaccharide degradation potential of *Aspergillus nidulans* and comparison to *Aspergillus niger* and *Aspergillus oryzae*. Fungal Genet Biol.

[12] Pérez S, Bertoft E (2010). The molecular structures of starch components and their contribution to the architecture of starch granules: a comprehensive review. Starch/Staerke.

[13] Nekiunaite L, Arntzen M, Svensson B, Vaaje-Kolstad G, Abou Hachem M (2016). Lytic polysaccharide monooxygenases and other oxidative enzymes are abundantly secreted by *Aspergillus nidulans* grown on different starches. Biotechnol Biofuels.

[14] Streb S, Zeeman SC (2012). Starch metabolism in arabidopsis..

[15] Hizukuri S, Takeda Y, Yasuda M, Suzuki A (1981). Multi-branched nature of amylose and the action of debranching enzymes. Carbohydr Res.

[16] Levasseur A, Drula E, Lombard V, Coutinho PM, Henrissat B (2013). Expansion of the enzymatic repertoire of the CAZy database to integrate auxiliary redox enzymes. Biotechnol Biofuels.

[17] Christiansen C, Abou Hachem M, Janeček Š, Viksø-Nielsen A, Blennow A, Svensson B (2009). The carbohydrate-binding module family 20-diversity, structure, and function. FEBS J.

[18] Kaminskyj SGW (2001). Fundamentals of growth, storage, genetics and microscopy of *Aspergillus nidulans*. Fungal Genet Rep..

[19] Hansen BG, Salomonsen B, Nielsen MT, Nielsen JB, Hansen NB, Nielsen KF (2011). Versatile enzyme expression and characterization system for *Aspergillus nidulans*, with the *Penicillium brevicompactum* polyketide synthase gene from the mycophenolic acid gene cluster as a test case. Appl Environ Microbiol.

[20] Gow NAR, Latge J-P, Munro CA (2017). The fungal cell wall: structure, biosynthesis, and function. Microbiol Spectr..

[21] Forsberg Z, Sørlie M, Petrović D, Courtade G, Aachmann FL, Vaaje-Kolstad G (2019). Polysaccharide degradation by lytic polysaccharide monooxygenases. Curr Opin Struct Biol.

[22] Tandrup T, Frandsen KEH, Johansen KS, Berrin JG, Leggio L (2018). Recent insights into lytic polysaccharide monooxygenases (LPMOs). Biochem Soc Trans.

[23] Garajova S, Mathieu Y, Beccia MR, Bennati-Granier C, Biaso F, Fanuel M (2016). Single-domain flavoenzymes trigger lytic polysaccharide monooxygenases for oxidative degradation of cellulose. Sci Rep..

[24] Villares A, Moreau C, Bennati-Granier C, Garajova S, Foucat L, Falourd X (2017). Lytic polysaccharide monooxygenases disrupt the cellulose fibers structure. Sci Rep..

[25] Crouch LI, Labourel A, Walton PH, Davies GJ, Gilbert HJ (2016). The contribution of non-catalytic carbohydrate binding modules to the activity of lytic polysaccharide monooxygenases. J Biol Chem.

[26] Chalak A, Villares A, Moreau C, Haon M, Grisel S, D’Orlando A (2019). Influence of the carbohydrate-binding module on the activity of a fungal AA9 lytic polysaccharide monooxygenase on cellulosic substrates. Biotechnol Biofuels.

[27] Nekiunaite L, Isaksen T, Vaaje-Kolstad G, Abou Hachem M (2016). Fungal lytic polysaccharide monooxygenases bind starch and β-cyclodextrin similarly to amylolytic hydrolases. FEBS Lett.

[28] Vu VV, Hangasky JA, Detomasi TC, Henry SJW, Ngo ST, Span EA (2019). Substrate selectivity in starch polysaccharide monooxygenases. J Biol Chem.

[29] Nielsen JB, Nielsen ML, Mortensen UH (2008). Transient disruption of non-homologous end-joining facilitates targeted genome manipulations in the filamentous fungus *Aspergillus nidulans*. Fungal Genet Biol.

[30] Nødvig CS, Hoof JB, Kogle ME, Jarczynska ZD, Lehmbeck J, Klitgaard DK, et al. Efficient oligo nucleotide mediated CRISPR-Cas9 gene editing in Aspergilli. Fungal Genet Biol. Academic Press Inc.; 2018;115:78–89. 10.1016/j.fgb.2018.01.004 29325827

[31] Cove DJ (1966). The induction and repression of nitrate reductase in the fungus *Aspergillus nidulans*. Biochim Biophys Acta.

[32] Nørholm MHH (2010). A mutant Pfu DNA polymerase designed for advanced uracil-excision DNA engineering. BMC Biotechnol.

[33] Nielsen ML, Albertsen L, Lettier G, Nielsen JB, Mortensen UH (2006). Efficient PCR-based gene targeting with a recyclable marker for Aspergillus nidulans. Fungal Genet Biol.

[34] Nødvig CS, Nielsen JB, Kogle ME, Mortensen UH (2015). A CRISPR-Cas9 system for genetic engineering of filamentous fungi. PLoS ONE.

[35] Zeuner B, Muschiol J, Holck J, Lezyk M, Gedde MR, Jers C (2018). Substrate specificity and transfucosylation activity of GH29 α-l-fucosidases for enzymatic production of human milk oligosaccharides. New Biotechnol..

