# Supplementary material for: Loss of AA13 LPMOs impairs degradation of resistant starch and reduces the growth of Aspergillus nidulans
Source: Biotechnol Biofuels. 2020 Aug 5;13:135. doi: 10.1186/s13068-020-01775-z (PMC7405360; doi:10.1186/s13068-020-01775-z)
Supplement: Supplementary file 1 — Additional file 1: Table S1. List of strains used in the study. Table S2. Primers for generation and validation of the A. nidulans mutant strains. Fig. S1. Gene targeting and mutant-strain validation. Fig. S2. Growth of reference and AA13 gene deletion strains on glucose. Fig. S3. Growth of reference and AA13 gene deletion strains on soluble potato starch. Fig. S4. Growth of reference and AA13 gene deletion strains on resistant potato starch. Fig. S5. Biomass analysis of reference and AA13 gene deletion strains on resistant potato starch at day 5. Fig. S6. Principal component analysis of the proteomic data sets during growth on resistant starch. Fig. S7. Venn diagram of the significantly upregulated proteins during growth on resistant potato starch. [file 13068_2020_1775_MOESM1_ESM.pdf]

Additional File 1

Loss of AA13 LPMOs impairs degradation of resistant starch and reduces the growth of *Aspergillus nidulans*

Majid Haddad Momeni, Maria Louise Leth, Claus Sternberg, Erwin Schoof, Maike Wennekers Nielsen, Jesper Holck, Christopher T. Workman, Jakob Blæsbjerg Hoof, Maher Abou Hachem*

Department of Biotechnology and Biomedicine, Technical University of Denmark, Søltofts Plads, 2800 Kgs. Lyngby, Denmark

*Correspondence: maha@bio.dtu.dk

Content:

Table S1. List of strains used in the study
Table S2. Primers for generation and validation of the *A. nidulans* mutant strains
Fig. S1. Gene targeting and mutant-strain validation
Fig. S2. Growth of reference and AA13 gene deletion strains on glucose
Fig. S3. Growth of reference and AA13gene deletion strains on soluble potato starch
Fig. S4. Growth of reference and AA13 gene deletion strains on resistant potato starch
Fig. S5. Biomass analysis of reference and AA13 gene deletion strains on resistant potato starch at day 5.
Fig. S6. Principal component analysis of the proteomic data sets during growth on resistant starch
Fig. S7. Venn diagram of the significantly upregulated proteins during growth on resistant potato starch

Table S1. List of strains used in the study

Strain ID	Genotype[a]	Tool[b]	Growth[c]	Strain purpose
NID1	*argB2, pyrG89, veA1, nkuA*Δ	-	arg, ura, uri	Host strain for gene targeting
NID731	*veA1, nkuA*Δ	-	none	Reference strain for analysis
NID2018	*argB2, pyrG89, veA1, nkuA*Δ*, aasB*Δ*, aasA*Δ*::AfpyrG*	GT	arg	Double AA13-gene deletion
NID2114	*argB2, pyrG89, veA1, nkuA*Δ*, aasA*Δ*::AfpyrG*	GT	arg	Single AA13-gene deletion
NID2115	*argB2, pyrG89, veA1, nkuA*Δ*, aasB*Δ*::AfpyrG*	GT	arg	Single AA13-gene deletion
NID2428	*pyrG89, veA1, nkuA*Δ, *aasA*Δ::*AfpyrG*	CC	none	Based on NID2114
NID2429	*pyrG89, veA1, nkuA*Δ, *aasB*Δ, *aasA*Δ::*AfpyrG*	CC	none	Based on NID2018
NID2438	*pyrG89, veA1, nkuA*Δ, *aasB*Δ::*AfpyrG*	CC	none	Based on NID2115

[a] *aasA* = AN5463, *aasB* = AN6103

[b] Engineering tool applied to construct strain in this study; GT is gene targeting and CC is CRISPR/Cas9-mediated gene editing

[c] Growth requirements may be arginine, uracil and uridine. Final strains for analysis have no growth requirements

Table S2. Primers for generation and validation of the *A. nidulans* mutant strains

Code	Usage	Locus[a]	Sequence
<u>For gene-targeting substrate construction</u>			
A1-F	*aasA*-Up fragment	*aasA*	GGGTTTAAUGTCGAAGGAGCAAAAGATCGGTG
A2-R	*aasA*-Up fragment	*aasA*	GGACTTAAUGGTGAGCTGTTTTCAGATTGAAGCG
A3-F	*aasA*-Dw fragment	*aasA*	GGCATTAAUGTGGATGAAGATCTGGAGAG
A4-R	*aasA*-Dw fragment	*aasA*	GGTCTTAAUGGAGACTCTGAGAGGGTGATG
B1-F	*aasB*-Up fragment	*aasB*	GGGTTTAAUGAGAAGCGCGAGGAAGGG
B2-R	*aasB*-Up fragment	*aasB*	GGACTTAAUGATGAGTTGCTGTGCTGCTTG
B3-F	*aasB*-Dw fragment	*aasB*	GGCATTAAUCGGTGGAGTGTGCAGAAAG
B4-R	*aasB*-Dw fragment	*aasB*	GGTCTTAAUGTGGGGAGTGAGTCAAGGG
<u>Gene-deletion analysis (see Figure S1)</u>			
D1-F	Reaction type !	*aasA*	CCTCACCTTATCGTCACGTTCC
D2-F	Reaction type !	*aasB*	CCCACGAGCCCTAAATTCAAC
D3-R	Reaction type !	*AfpyrG*	GGAAGAGAGGTTCACACC
D4-F	Reaction type II	*aasA*	CATCCCCAATGCTCAAGACCC
D5-R	Reaction type II	*aasA*	CTTGGCTCAGAGCAAGCGC
D6-F	Reaction type II	*aasB*	GAGATACTCGGAGAGAGAGCG
D7-R	Reaction type II	*aasB*	GCGTACATGTGACATGGGAATG
<u>Gene-editing oligo for restoring *argB*</u>			
GE-9[b]	Repair substrate	*argB2*	TCCTTGGGAAAACTGTGGCCATGATCTTCAGCAAAC GAAGCACGAGGACAAGGGTATCTACAGAAGGGGCC GTTGTGCAGATGGGAGGTC[A]

[a] IDs for targeted loci are *aasA*=AN5463 and *aasB*=AN6103

[b] GE-Oligo9 to be used in transformation with CRISPR vector pFC907, both from Nødvig CS, Hoof JB, Kogle ME, Jarczynska ZD, Lehmbeck J, Klitgaard DK, Mortensen UH: **Efficient oligo nucleotide mediated CRISPR-Cas9 gene editing in Aspergilli**. *Fungal Genet Biol* 2018, **115**:78-89.

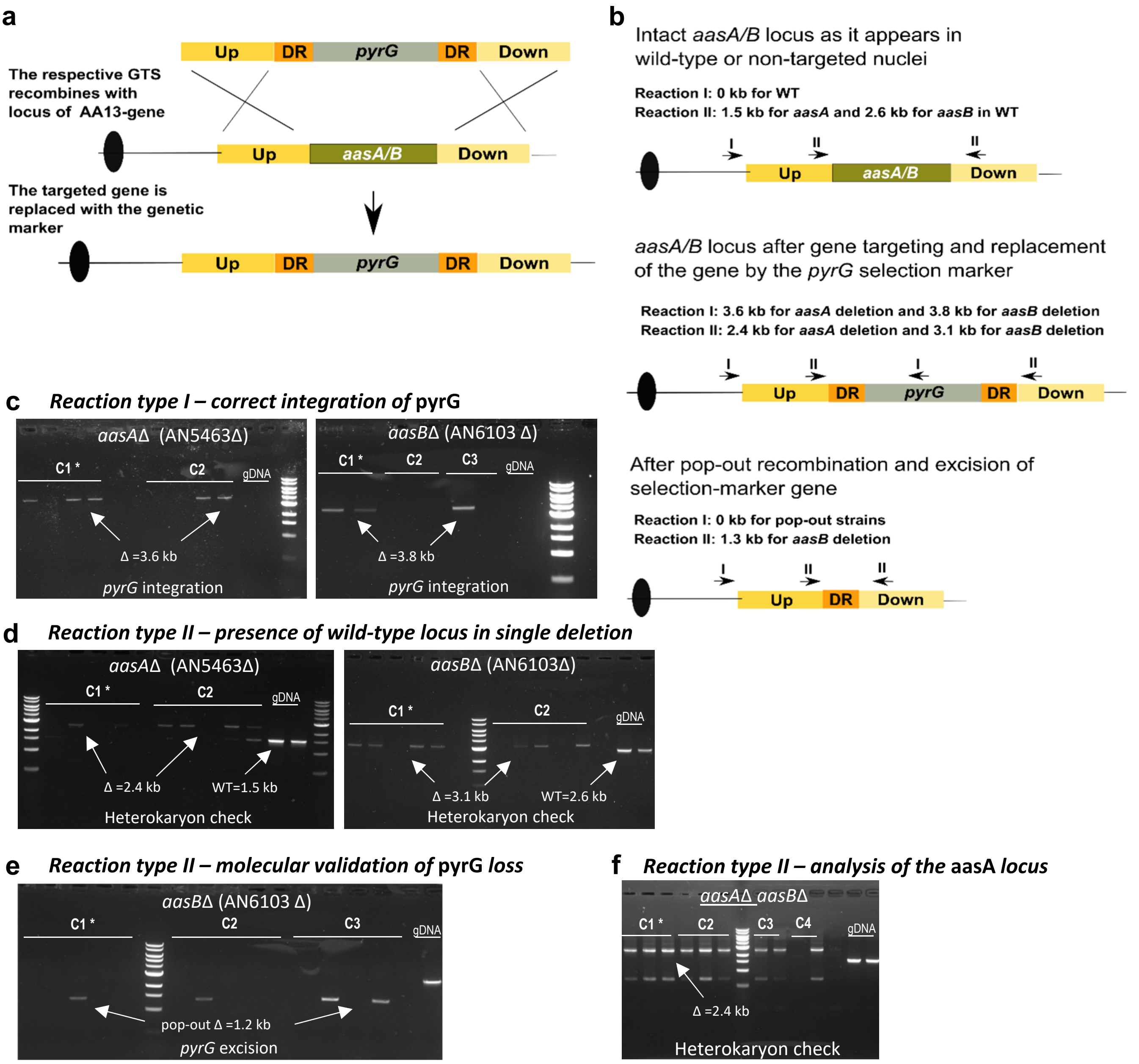

Fig. S1. Gene targeting and mutant-strain validation. **a** Target-gene replacement with a selection marker by homologous recombination. **b** Mutant-strain evaluation by diagnostic PCR. Top illustrates the *aasA/aasB* loci, middle shows the replacement of *aasA* or *aasB* by the heterologous *pyrG* (*AfpyrG*). After pop-out recombination of the *pyrG* marker, the remaining sequence patch from one of the direct repeats is depicted in the bottom. Primers for reaction type I anneal outside the targeted region and inside the selection marker, thus a correct-sized band amplified by PCR, indicates successful gene targeting; Primers for reaction type II evaluates presence of remaining nuclei in the colonies of the intact versions of the targeted gene. For diagnostic tissue-PCRs, 2-5 reactions including the same strains are typically analyzed depending on the efficiency. Figure is not to scale. See Table S2 for the primers used in the tissue-PCR analysis. **c** Diagnostic tissue-PCR for verifying single deletions of *aasA* (left) and *aasB* (right) using reaction type I. These analyses showed that two independent restreaked transformants (colony 1 (C1) and colony 2(C2)) had *pyrG* inserted in the targeted loci with respective bands of 3.6 and 3.8 kb. The colony with a star was used for further work and analysis. The control reactions in the reference strain using genomic DNA gave no corresponding band due to the absence of the heterologous *pyrG*. **d** The check for heterokaryotic (untargeted) single-deletion strains is carried out with PCR-reaction type 2. The single deletions were analyzed, *aasA* (left) and *aasB* (right). All reactions showed a clear distinction between mutant and wild-type loci, and the expected sizes for the specific loci for both scenarios are indicated. Only in one case, for C2 in the left panel we observed a heterokaryon. **e** The *aasB* single deletion was cultivated on 5-FOA containing growth medium, and resulting colonies were restreaked and analyzed using reaction type II. **f** The double-deletion strain was constructed in the *aasB*Δ background, by targeting *aasA,* and transformants were analyzed and verified in reaction type II as in **d** (left).

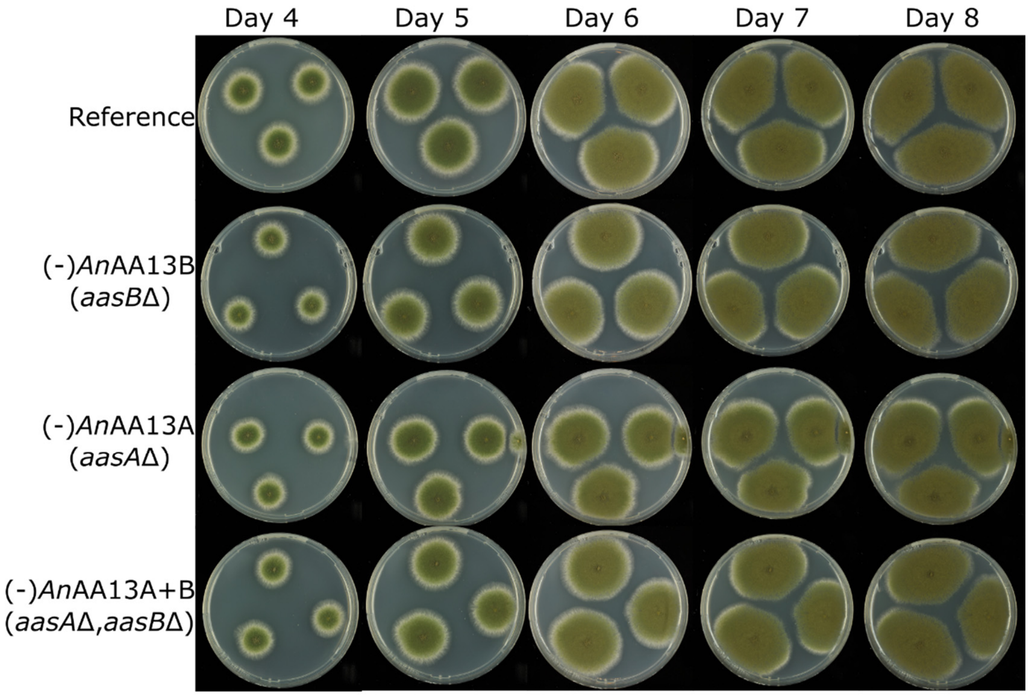

Fig. S2. Growth of reference and AA13 gene deletion strains on glucose. The minimal media plates are supplemented with 1% (w/v) carbohydrate substrates.

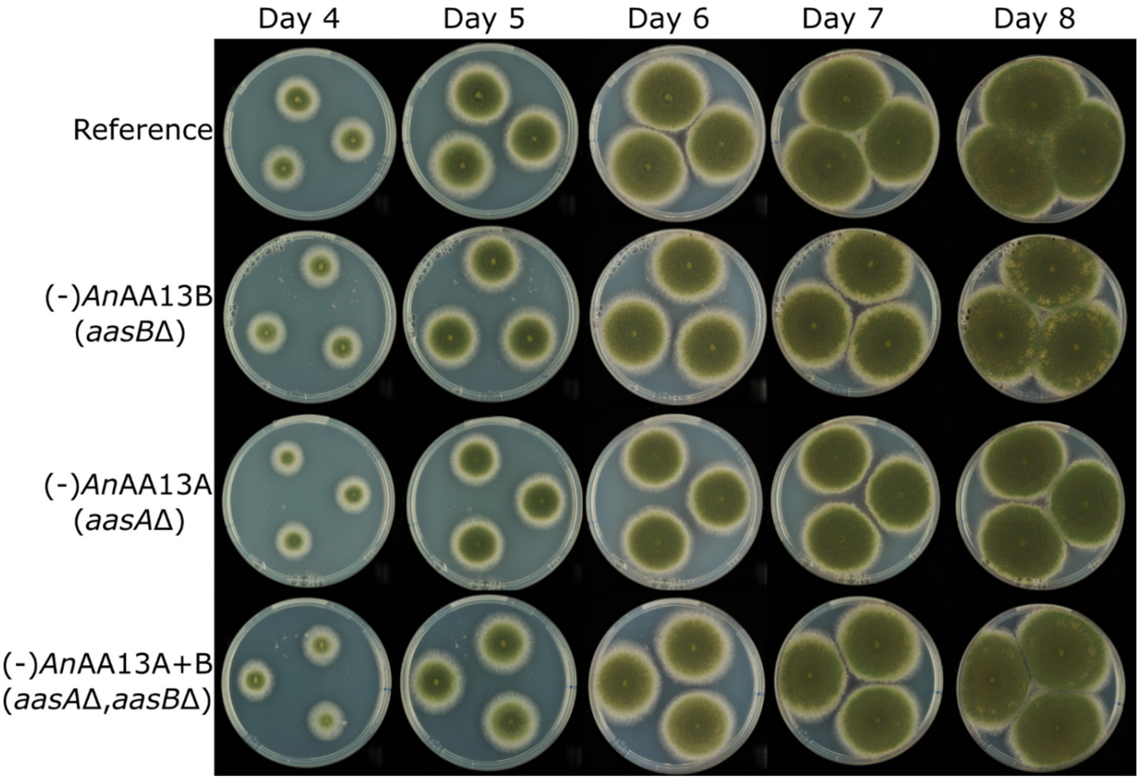

Fig. S3. Growth of reference and AA13-gene deletion strains on soluble potato starch. The minimal media plates are supplemented with 1% (w/v) carbohydrate substrates.

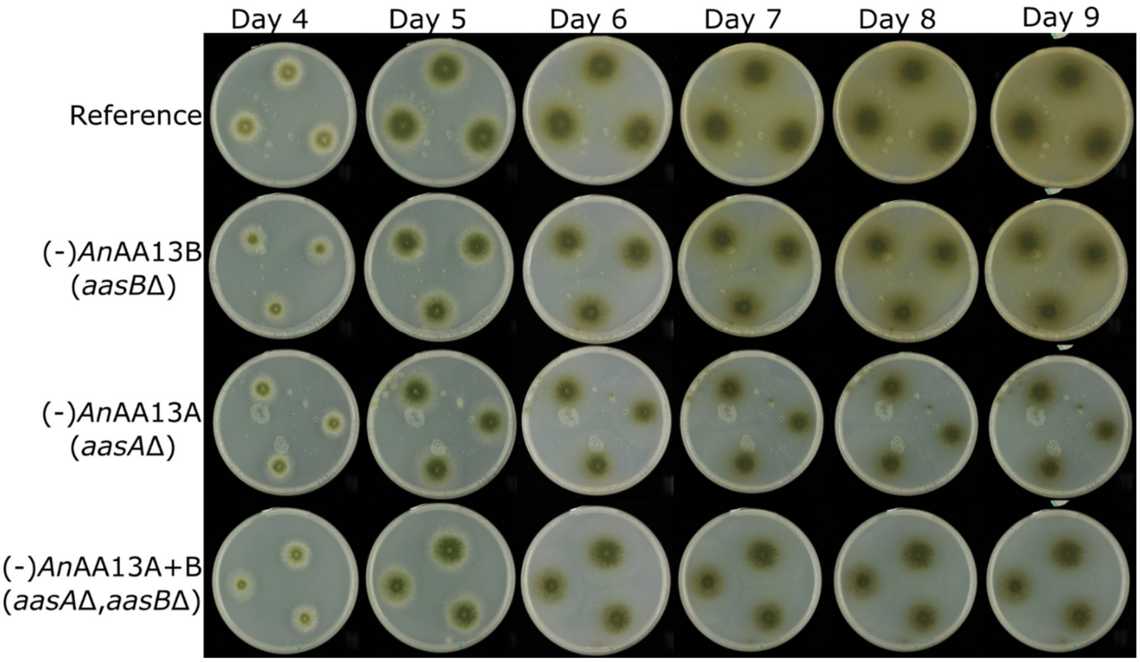

Fig. S4. Growth of reference and AA13 gene deletion strains on resistant potato starch. The minimal media plates are supplemented with 1% (w/v) carbohydrate substrates.

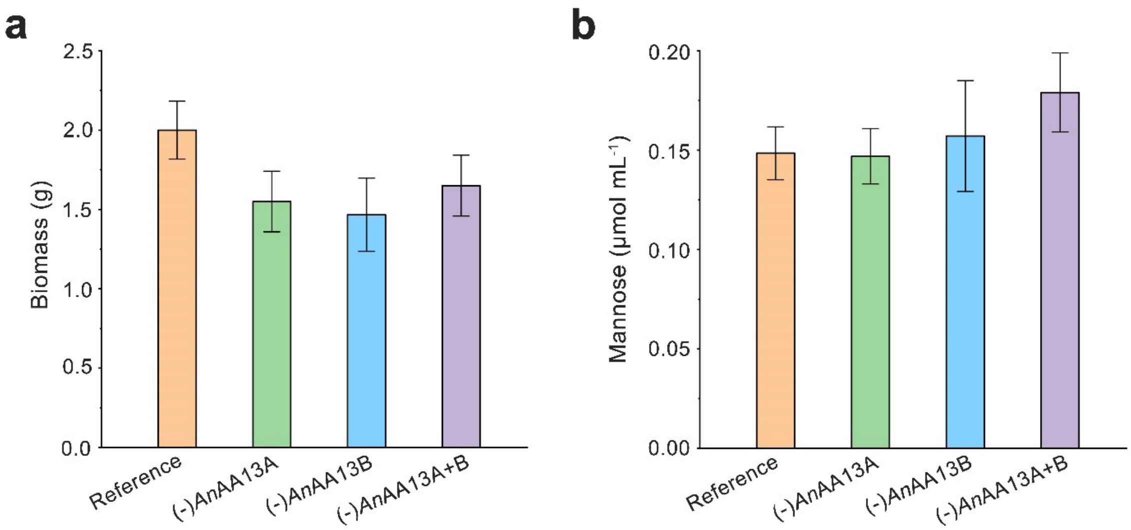

Fig. S5. Biomass analysis of the reference and KO *A. nidulans* strains at day 5. **a** Weight of wet biomass scraped from a cloth filter at the harvest of the liquid culture supplemented with 1% (w/v) resistant potato starch. **b** Mannose content analysed after overnight acid hydrolysis and analysis by HPAEC-PAD of the samples in (a). The mannose content is measured as a proxy of biomass, as mannan is structural component of the fungal cell wall. Both measurements are performed in four biological replicates.

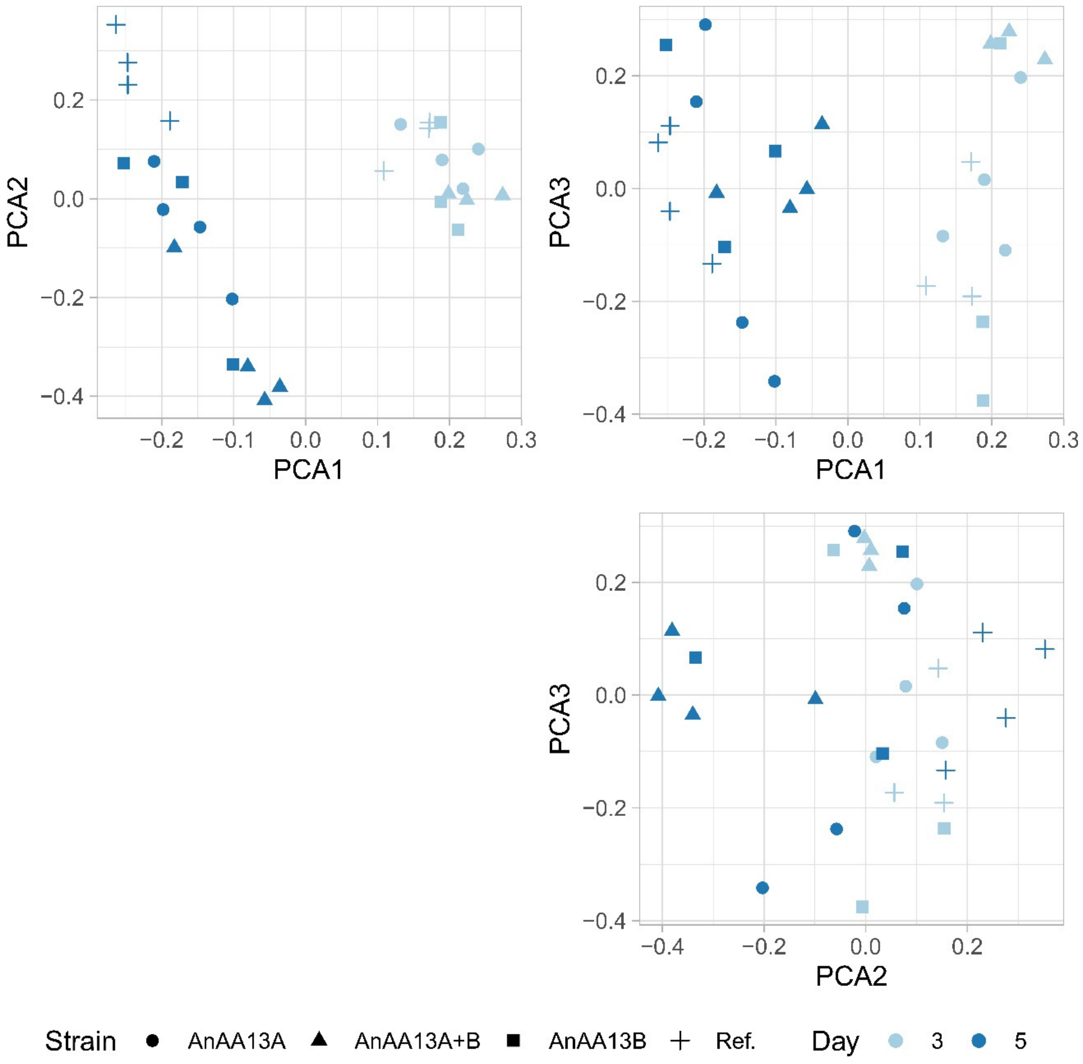

Fig. S6. Principal component analyses of the proteomic data sets from biological replicates of the reference and the AA13 gene deletion strains. The plot were generated in R statistical computing software.

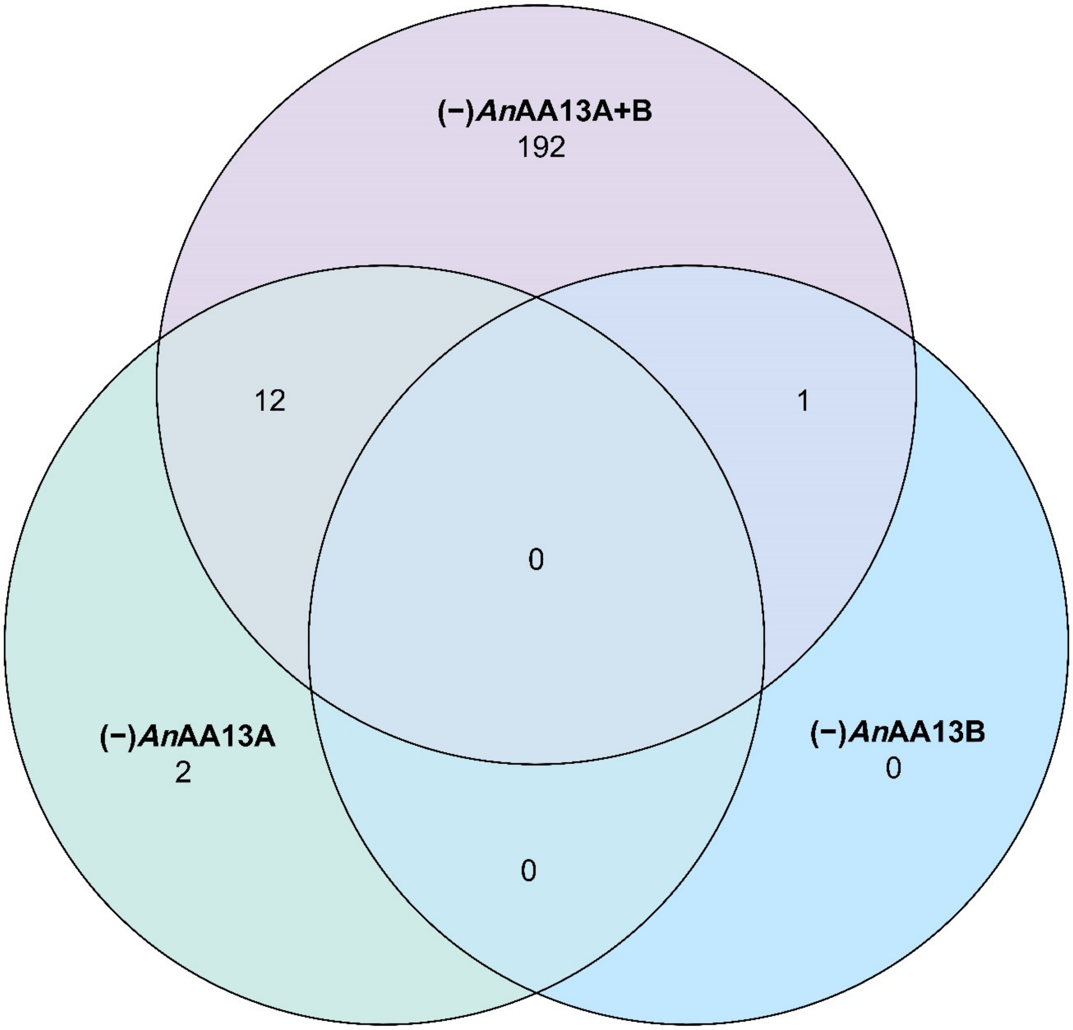

Fig. S7. Venn diagram of the significantly regulated proteins for AA13 gene deletion strains (Log_2 fold change >2, P≤0.05).
